# Draft Genome Sequence of *Frankia* sp. Strain Thr, a Nitrogen-Fixing Actinobacterium Isolated from the Root Nodules of *Casuarina cunninghamiana* Grown in Egypt

**DOI:** 10.1128/genomeA.00493-14

**Published:** 2014-05-22

**Authors:** Sheldon G. Hurst, Rediet Oshone, Faten Ghodhbane-Gtari, Krystalynne Morris, Feseha Abebe-Akele, W. Kelley Thomas, Amir Ktari, Karima Salem, Samira Mansour, Maher Gtari, Louis S. Tisa

**Affiliations:** a University of New Hampshire, Durham, New Hampshire, USA; b Laboratoire Microorganismes et Biomolécules Actives, Université Tunis El Manar (FST) & Université Carthage (INSAT), Campus Universitaire, Tunis, Tunisia; c Botany Department, Faculty of Science, Suez Canal University, Ismailia, Egypt

## Abstract

Nitrogen-fixing actinobacteria of the genus *Frankia* are symbionts of woody dicotyledonous plants termed actinorhizal plants. We report here a 5.3-Mbp draft genome sequence for *Frankia* sp. stain Thr, a nitrogen-fixing actinobacterium isolated from root nodules of *Casuarina cunninghamiana* collected in Egypt.

## GENOME ANNOUNCEMENT

Among the *Actinobacteria*, the genus *Frankia* is well known for its facultative lifestyle as a plant symbiont of dicotyledonous plants, termed actinorhizal plants, and as a free-living soil dweller (1–3). Actinorhizal plants are ecologically important pioneer community plants that are found worldwide under a broad range of ecological and environmental conditions (4). The symbiosis allows actinorhizal plants to colonize harsh environmental terrains. Based on molecular phylogenetic analysis, four major clusters within the genus are recognized (5–9), and genomes for representatives from each cluster have been sequenced (10–16). Cluster I contains two subclusters: one subcluster (cluster Ia) represents *Frankia* strains with the ability to infect a wide range of host plants, including members of the *Betulaceae* and *Myricaceae* families, and the other subcluster (cluster Ib) contains strains limited to *Casuarina* and *Allocasuarina* host plants.

The fast growing and highly tolerant trees from the family *Casuarinaceae* have been used worldwide as windbreaks, dune stabilizers, fuel wood, and in soil regeneration (17). In North Africa, these actinorhizal plants are grow well under the harsh conditions. *Frankia* sp. strain Thr was isolated from root nodules of *Casuarina cunninghamiana* growing in Egypt and effectively reinfects its original host plant, one of the *Casuarina* spp. (18). *Frankia* sp. strain Thr has been used extensively in infection studies and is well characterized for its host plant interactions. Presently, two genomes from *Frankia* cluster Ib *Casuarina*-infecting strains are available (10, 16). *Frankia* sp. strain Thr was sequenced to increase our understanding of this subcluster Ib with restricted host plant range and to provide information about its potential ecological roles and interaction with actinorhizal plants.

The draft genome of *Frankia* sp. strain Thr was generated at the Hubbard Genome Center (University of New Hampshire, Durham, NH) using Illumina technology (19) techniques. A standard Illumina shotgun library was constructed and sequenced using the Illumina HiSeq 2000 platform, which generated 19,189,718 reads (260-bp insert size) totaling 1,824.9 Mbp. The Illumina sequence data were assembled using the CLC Genomics Workbench (6.5.1) and AllPaths-LG (version r41043) (20). The final draft assembly contained 171 contigs, with an *N*_50_ of 71.6 kb. The total size of the genome is 5.3 Mbp, and the final assembly is based on 1,666.1 Mb of Illumina draft data, providing an average 248× coverage of the genome.

The high-quality draft genome of *Frankia* sp. strain Thr was resolved to 171 contigs consisting of 5,309,833 bp, with a G+C content of 69.95%. The assembled *Frankia* sp. strain Thr genome was annotated via the Integrated Microbial Genomes (IMG) platform developed by the Joint Genome Institute, Walnut Creek, CA, USA (21), and resulted in 4,805 candidate protein-coding genes, 46 tRNA genes, and 2 rRNA regions.

### Nucleotide sequence accession numbers.

This whole-genome shotgun project has been deposited at DDBJ/EMBL/GenBank under the accession no. JENI00000000. The version described in this paper is version JENI01000000.
